# Cancer reversion with oocyte extracts is mediated by cell cycle arrest and induction of tumour dormancy

**DOI:** 10.18632/oncotarget.24664

**Published:** 2018-03-23

**Authors:** Norazalina Saad, Ramiro Alberio, Andrew D. Johnson, Richard D. Emes, Tom C. Giles, Philip Clarke, Anna M. Grabowska, Cinzia Allegrucci

**Affiliations:** ^1^ School of Veterinary Medicine and Science, University of Nottingham, Sutton Bonington Campus, Loughborough, LE12 5RD, UK; ^2^ Institute of Bioscience, Universiti Putra Malaysia, 43400 UPM Serdang, Selangor, Malaysia; ^3^ School of Biosciences, Sutton Bonington Campus, Loughborough, LE12 5RD, UK; ^4^ School of Life Sciences, University of Nottingham, QMC, Nottingham, NG7 2UH, UK; ^5^ Advanced Data Analysis Centre (ADAC), University of Nottingham, Sutton Bonington Campus, Loughborough, LE12 5RD, UK; ^6^ Cancer Biology, Division of Cancer and Stem Cells, School of Medicine, University of Nottingham, QMC, Nottingham, NG7 2UH, UK

**Keywords:** reprogramming, tumour reversion, dormancy, breast cancer, axolotl oocytes

## Abstract

Inducing stable control of tumour growth by tumour reversion is an alternative approach to cancer treatment when eradication of the disease cannot be achieved. The process requires re-establishment of normal control mechanisms that are lost in cancer cells so that abnormal proliferation can be halted. Embryonic environments can reset cellular programmes and we previously showed that axolotl oocyte extracts can reprogram breast cancer cells and reverse their tumorigenicity. In this study, we analysed the gene expression profiles of oocyte extract-treated tumour xenografts to show that tumour reprogramming involves cell cycle arrest and acquisition of a quiescent state. Tumour dormancy is associated with increased P27 expression, restoration of RB function and downregulation of mitogen-activated signalling pathways. We also show that the quiescent state is associated with increased levels of H4K20me3 and decreased H4K20me1, an epigenetic profile leading to chromatin compaction. The epigenetic reprogramming induced by oocyte extracts is required for RB hypophosphorylation and induction of P27 expression, both occurring during exposure to the extracts and stably maintained in reprogrammed tumour xenografts. Therefore, this study demonstrates the value of oocyte molecules for inducing tumour reversion and for the development of new chemoquiescence-based therapies.

## INTRODUCTION

Cancer is a disease characterised by abnormal cell proliferation and first line treatments aim to eradicate tumour growth and metastatic spread. Novel therapies are being developed to specifically target molecular mechanisms at the basis of tumorigenesis to affect oncogenic pathways [1, 2]. However, they do not target all the molecular alterations that contribute to the complexity of the disease and they often result in drug resistance by allowing regrowth of tumour clones that do not respond to treatment [3]. Another approach to the development of targeted therapies is studying the mechanisms of tumour reversion, a biological process by which tumour cells lose or escape their malignant phenotype [4]. Tumour reversion can be induced by environmental cues with cells losing their malignant state following induction of growth arrest, apoptosis or differentiation [4]. Different models of tumour reversion have been explored and critical genes include a number already identified as targets of established drugs that could be repurposed for cancer treatment [5]. However, because tumour reversion involves multiple genes [6], a full understanding of its complexity still needs to be realised.

Among tumour reversion models, embryonic environments present unique opportunities due to their intrinsic ability to epigenetically program cellular states during development [7]. Embryos of different species have been used to suppress tumorigenicity of cancer cells and this approach was extended to embryonic stem cell extracts [8]. Indeed, teratocarcinoma cells can be reprogrammed when injected into blastocysts and contribute to normal tissue of chimeric mice [9]. In addition, injections into embryos of different species including zebrafish, chicken and mouse have been shown to reduce tumorigenicity and metastasis of melanoma cells [10–12]. Studies based on tumour reversion with these systems demonstrated that cancer cells retain a level of plasticity and are able to respond to embryonic signals that can induce loss of malignant phenotypes and/or growth arrest. Cancer cell plasticity can also be exploited for tumour reversion by nuclear reprogramming, an epigenetic process involving the change of one cellular state into another [13, 14]. Nuclear transfer experiments with somatic cells have shown that oocytes are able to reprogram somatic chromatin with remodelling of histone variants, histone modifications, DNA methylation and incorporation of oocyte-specific factors which actively participate in the reprogramming process [15–18]. The ability of oocytes to mediate tumour reversion has been further confirmed by *ex ovo* reprogramming experiments using extracts from amphibian and mammalian oocytes [19, 20]. Among amphibians, axolotls are unique experimental models because the molecular mechanisms regulating early development of axolotls and mammals are conserved [21–23]. Axolotl oocytes are very large and available in significant quantities, thus representing a unique experimental system for biochemical studies. We have previously introduced the value of axolotl oocyte extracts in tumour reversion and showed that they can reprogram breast cancer cells and suppress tumour growth *in vivo* [20].

In this study, we extended our investigation of tumour reversion with axolotl oocyte extracts by analysing the molecular profile of tumour-reverted mouse xenografts to show that oocyte-mediated reprogramming of breast cancer cells induces growth suppression by cell cycle arrest and induction of cellular dormancy. The growth arrest is associated with the upregulation of the cell cycle inhibitor P27, inhibition of RB phosphorylation and of key signalling pathways involved in cell proliferation. Growth arrested tumours demonstrated extensive epigenetic reprogramming with increased H4K20me3 and reduced H4K20me1, which are hallmarks of quiescence. Importantly, we show that the program of tumour reversion and tumour dormancy is initiated during the treatment with the oocyte extracts and is stably maintained in xenograft tumours over time.

## RESULTS

### Reprogrammed tumours show decreased proliferation associated with cell cycle arrest

**Figure 1:**
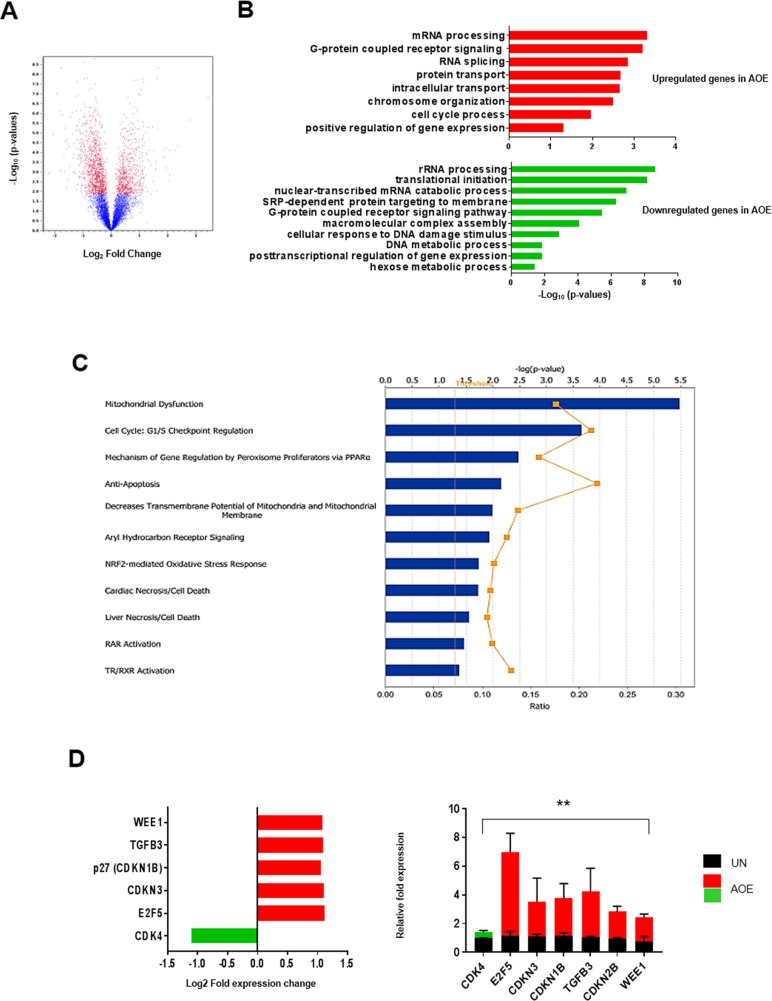
Gene expression analysis of AOE-reprogrammed tumour xenografts **(A)** Volcano plot of differentially expressed gene in AOE-treated versus untreated (UN) tumour xenografts. Differentially expressed genes were determined with a cut-off for adjusted p-value established at 0.05 and fold change value at ± 2 (n=6). Red dots show genes which are diffrerentially expressed above a 2-fold threshold. The part of the graft on the left of the zero point represents genes which are downregulated in AOE-treated tumours (1235), whereas the one on the right represents genes which are upregulated in AOE-treated tumours (741). **(B)** Bar plot ranking the enrichment score of GO biological processes for upregulated and downregulated genes in AOE-treated tumours compared to untreated. **(C)** Best match canonical pathway by IPA. Functional pathways are presented in descending order of significance (p<0.05, with threshold specified by yellow dotted line). The yellow graph line indicates the ratio between the numbers of genes from the dataset and the total number of genes involved in those pathways. **(D)** Fold change in gene expression as determined by microarray analysis (left) and TaqMan^®^ qRT-PCR (right). Quantitative RT-PCR results for each gene are presented as relative fold expression to *RPLP0* and the UN group used as calibrator (n=3). Relative fold expression levels were analysed by unpaired Student’s *t*-test. ^**^p< 0.01. Green and red bars indicate downregulated or upregulated genes in AOE, respectively.

Axolotl oocyte extracts (AOE) can reprogram breast cancer cells by reverting tumorigenicity *in vivo* [20]. In this study, we sought to identify the molecular mechanisms involved in reverting the tumour phenotype after treatment of breast cancer cells with AOE. We therefore determined the gene expression profile of tumour xenografts obtained from cells treated with AOE, as well as from untreated (UN) control cells. Microarray analyses revealed a total of 1976 differentially expressed genes, 741 and 1235 up-regulated and down-regulated in AOE-treated tumours, respectively (Figure 1A). Several biological processes associated with regulation of cell proliferation were identified by Gene Ontology (GO) analysis. RNA splicing and processing, chromosome organisation, cell cycle, intracellular transport, G coupled receptor signalling were significantly represented in the upregulated genes, whereas protein translation, protein targeting to membrane, rRNA processing, mRNA and cell metabolism were among the most significant in the downregulated genes (Figure 1B). The interaction between genes that were differentially expressed in AOE-treated tumours was also explored by mapping to gene networks identified by Ingenuity Pathway Analysis (IPA). Developmental disorder and metabolic disease, post-translational modification and cellular organisation, lipid metabolism, DNA replication and repair, cell death and cell growth/survival, and cell signalling were among the top networks identified. The top molecular and cellular functions also included protein synthesis, RNA post-translational modification, gene expression, cell growth and proliferation, and cell death and survival among the processes affected by the cancer cell reprogramming (Supplementary Figure 1A). IPA analysis showed Mitochondrial dysfunction and Cell cycle: G1/S checkpoint regulation as the most significant among the best match canonical pathways (Figure 1C). The latter pathway also demonstrated one of the highest gene ratios, indicating a high number of genes from the dataset represented in the total number of genes involved in this pathway. Consistent with this, genes involved in DNA replication and repair were found to be downregulated. These included members of the origin recognition complex assembly (*MCM2, MCM5, CDT1*), and DNA polymerases (*POLA1, POLD2, POLD4*) (Supplementary Figure 1B). Several important cell cycle regulators were found differentially expressed in the reprogrammed tumours, including *CDK4*, *E2F5*, and *CDKN1B* (*P27*), *WEE1, CDKN3* (Figure 1D). The canonical pathway analysis revealed additional cell cycle regulators that could be potentially involved, including antiproliferative BTG/TOB proteins, TGF-β, EIF2, mTOR Integrin and RHO signalling proteins (Supplementary Table 1). Differential expression of critical cell cycle genes was validated by via qRT-PCR, demonstrating the biological relevance of this pathway in the tumour-reverted phenotype (Figure 1D).

### Extract-induced cell cycle arrest is associated with increased CDKN1B (P27) expression and inactivation of RB function

**Figure 2:**
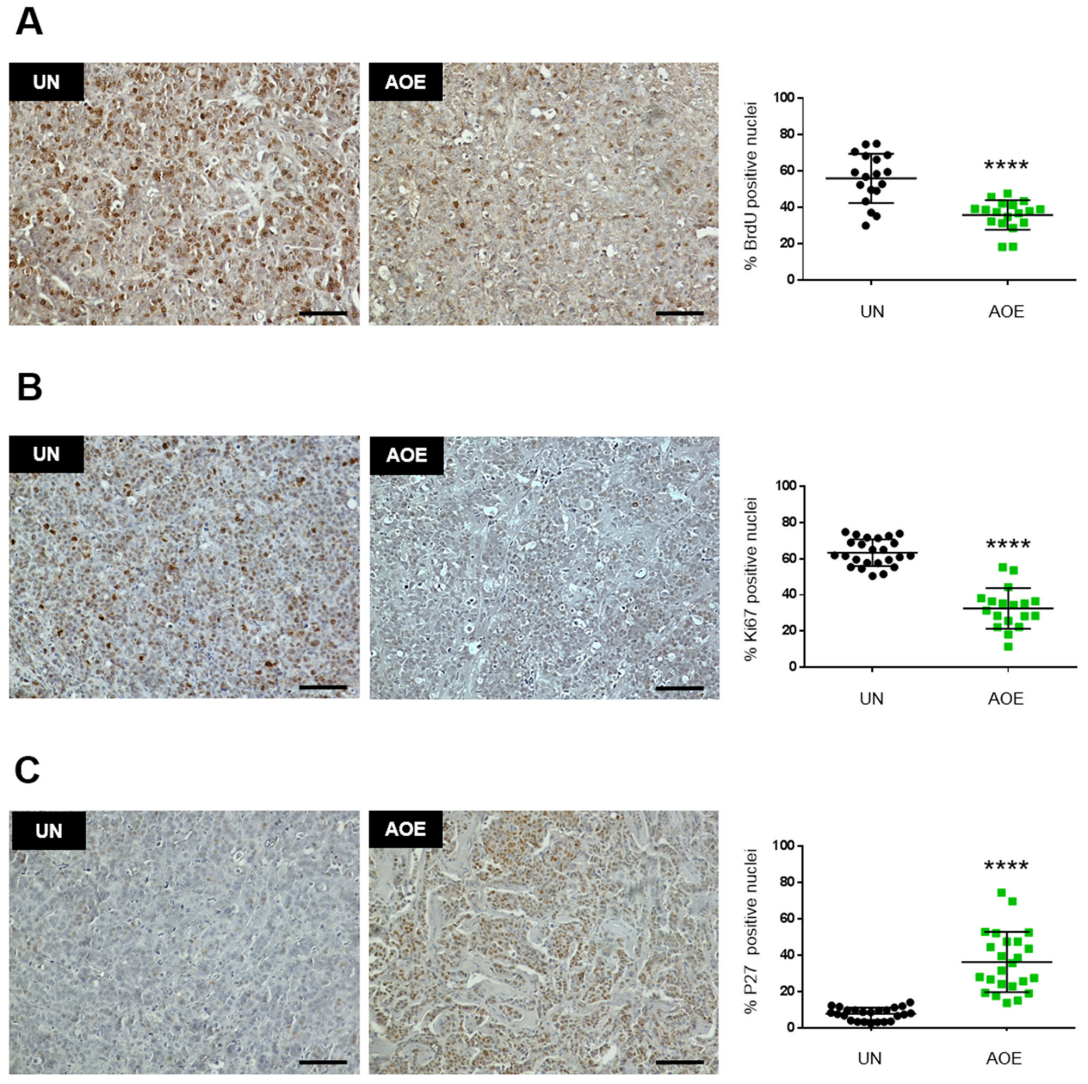
Quantification of BrdU, Ki67 and P27 staining in AOE-reprogrammed tumours **(A)** BrdU staining of UN and AOE-treated tumour xenografts. Scatter plot shows the % of positively stained nuclei (n= 3; 6 fields of view). Staining levels were analysed by nonparametric Mann-Whitney test. ^****^p<0.0001. Scale bar = 100μm. **(B)** Ki67 staining of UN and AOE-treated tumour xenografts. Scatter plot shows the % of positively stained nuclei (n= 3-4; 6 fields of view). Staining levels were analysed by nonparametric Mann-Whitney test. ^****^p<0.0001. Scale bar = 100μm. **(C)** P27 staining of UN and AOE-treated tumour xenografts. Scatter plot shows the % of positively stained nuclei (n= 4; 6 fields of view). Staining levels were analysed by nonparametric Mann-Whitney test. ^****^p<0.0001. Scale bar = 100μm.

To functionally validate the involvement of a cell cycle block we next conducted a BrdU labelling experiment of xenografts obtained from reprogrammed cancer cells (Supplementary Figure 2). Reprogrammed tumour xenografts showed reduced staining of the proliferation markers BrdU and Ki67, indicating a cell cycle block before S phase (Figure 2A, 2B). In addition, the same tumours showed increased expression of P27, which is consistent with the observed increased gene expression. Importantly, P27 staining was also nuclear in the reprogrammed tumours, suggesting its re-established function as tumour suppressor after the reprogramming (Figure 2C). As many pathways regulating G1-S cell cycle progression converge on the activity of the retinoblastoma tumour suppressor protein (RB), we next investigated the regulation of RB phosphorylation in reprogrammed tumours.

**Figure 3:**
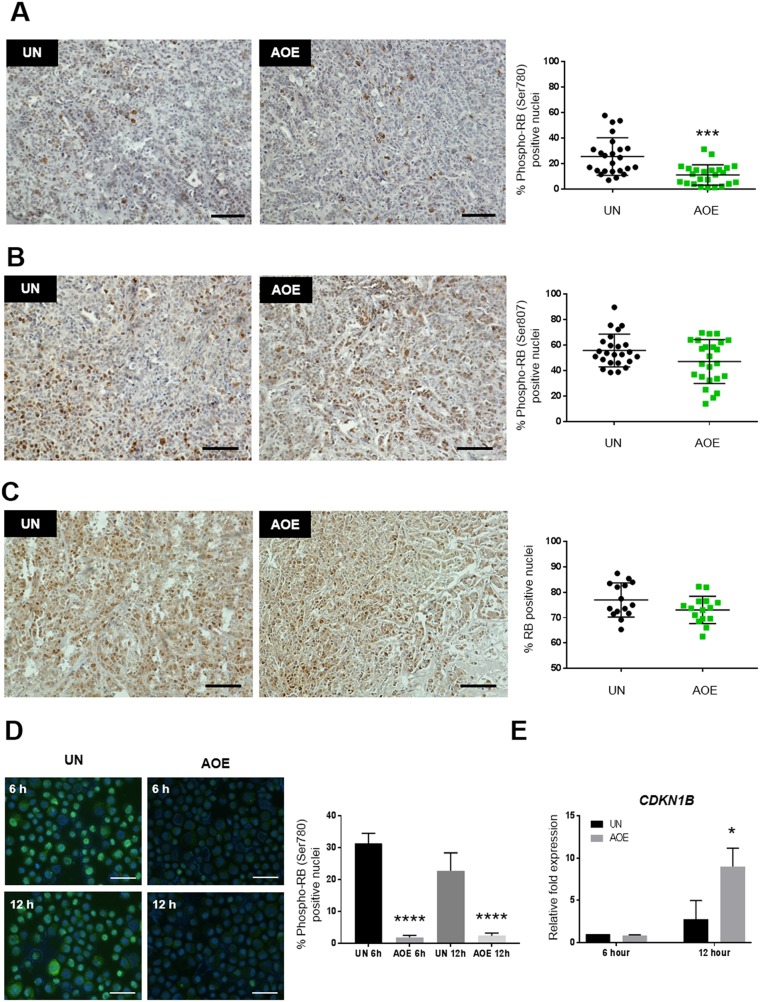
Quantification of RB activation and P27 expression in AOE-reprogrammed tumours and cancer cells **(A)** RB (Ser780) phosphorylation staining of UN and AOE-treated tumour xenografts. Scatter plot shows the % of positively stained nuclei (n= 4; 6 fields of view). Staining levels were analysed by nonparametric Mann-Whitney test. ^***^p<0.001. Scale bar = 100μm. **(B)** RB (Ser807) phosphorylation staining of UN and AOE-treated tumour xenografts. Scatter plot show the % of positively stained nuclei (n= 4; 6 fields of view). Staining levels were analysed by nonparametric Mann-Whitney test. p=0.1472. Scale bar = 100μm. **(C)** Total RB staining of UN and AOE-treated tumour xenografts. Scatter plot show the % of positively stained nuclei (n= 3; 5 fields of view). Staining levels were analysed by nonparametric Mann-Whitney test. p=0.1485. Scale bar = 100μm. **(D)** RB (Ser780) phosphorylation staining of UN and AOE-treated cells after 6 hours and 12 hours reprogramming. Scatter plot shows the % of positively stained nuclei (n= 2; 5 fields of view). Staining levels were analysed by One-way Anova followed by Bonferroni’s multiple comparisons test. ^****^p<0.0001. Scale bar = 50μm. **(E)** Fold change in P27 (*CDKN1B*) gene expression as determined by TaqMan^®^ qRT-PCR. Results are presented as relative fold expression to *RPLP0* and the UN group used as calibrator (n=3). Relative fold expression levels were analysed by Two-way Anova followed by Bonferroni’s multiple comparisons test. ^*^p< 0.05.

Consistent with cell cycle arrest and the inhibition of CDK activity, reprogrammed tumours showed reduced phosphorylation of RB at Ser780 (Figure 3A), but not at Ser807 or altered expression of total RB levels (Figure 3B, 3C). Phosphorylation of RB at Ser795 was not detected in either untreated or untreated xenografts (Supplementary Figure 3A). Hypo-phosphorylation of RB at Ser780 is consistent with the observed decreased CDK4 expression and the predicted negative z-score of the EIF2 canonical signalling pathway. These results further indicate a growth arrest phenotype resulting from active RB and inhibition of the progression through S and G2/M phases of the cell cycle (Supplementary Table 1).

In order to understand whether a cell cycle block might be an immediate response to extract treatment, we measured RB phosphorylation and P27 expression in treated cells at 6 hours and 12 hours after reprogramming, the latter representing the same time point at which the cells were injected in immunocompromised mice. Phosphorylation of RB and its nuclear localisation was reduced by 6 hours and this was maintained at low levels up to 12 hours after extract treatment (Figure 3D). On the other hand, expression of *CDKN1B (P27)* did not increase immediately after the extract treatment (6 hours), but it was induced by 12 hours after reprogramming (Figure 3E). These results indicate that the induction of cell cycle arrest occurs during the reprogramming process in oocyte extracts and it is stably maintained in treated tumour xenografts.

### Reprogrammed tumours show reduced MAPK signalling

In order to elucidate the contribution of cell cycle extrinsic regulation in oocyte-mediated cancer reprogramming, several signalling pathways involved in cell proliferation were analysed.

**Figure 4:**
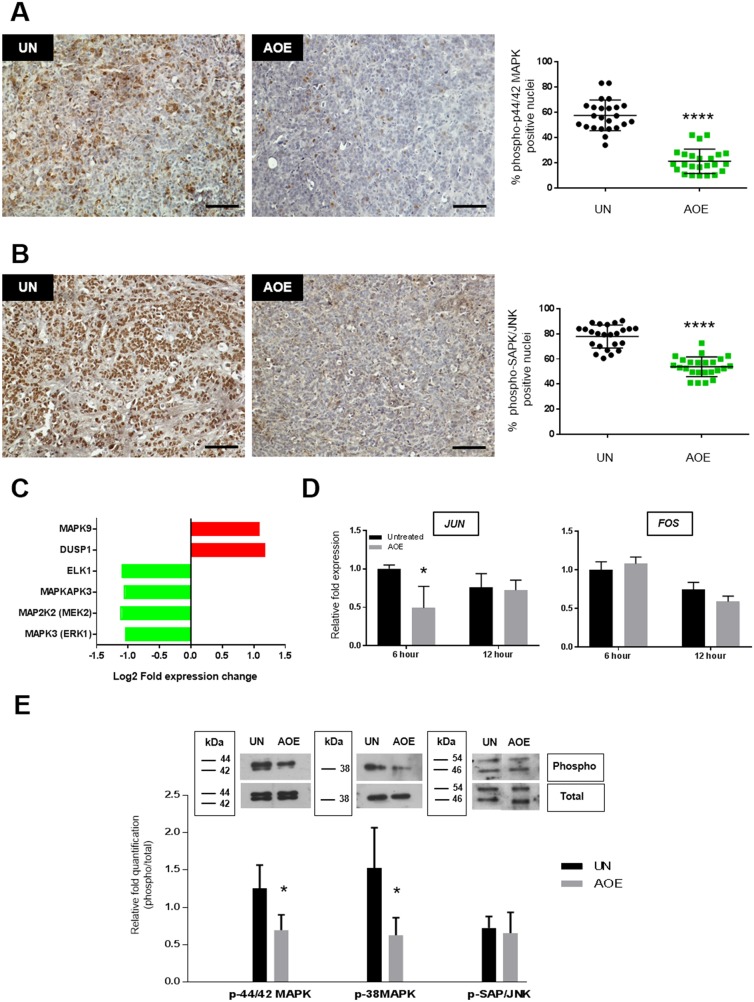
Quantification of p44/42 MAPK, SAPK/JNK and p38MAPK activation in AOE-reprogrammed tumours and cancer cells **(A)** p44/42 MAPK phosphorylation staining of UN and AOE-treated tumour xenografts. Scatter plot shows the % of positively stained nuclei (n= 4; 6 fields of view). Staining levels were analysed by nonparametric Mann-Whitney test. ^****^p<0.0001. Scale bar = 100μm. **(B)** SAPK/JNK phosphorylation staining of UN and AOE-treated tumour xenografts. Scatter plot show the % of positively stained nuclei (n= 4; 6 fields of view). Staining levels were analysed by nonparametric Mann-Whitney test. ^****^p<0.0001. Scale bar = 100μm. **(C)** Fold change in gene expression as determined by microarray analysis. **(D)** Fold change in *JUN* and *FOS* gene expression as determined by TaqMan^®^ qRT-PCR. Results are presented as relative fold expression to *RPLP0* and the UN group used as calibrator (n=3). Relative fold expression levels were analysed by Two-way Anova followed by Bonferroni’s multiple comparisons test. ^*^p< 0.05. **(E)** Western blotting analysis of p44/42 MAPK, SAPK/JNK and p38MAPK phosphorylation of cells treated with AOE for 6 hours. Relative quantification of phosphorylation was calculated as ratio between the levels of phosphorylated and total protein signals. Relative quantification was analysed by unpaired Student’s *t*-test. ^*^p< 0.05.

Reprogrammed tumours demonstrated reduced phosphorylation of p44/42 MAPK and SAPK/JNK (Figure 4A, 4B), whereas no activation of p38 MAPK was observed in either untreated or reprogrammed tumours (Supplementary Figure 3B). The downregulation of ERK signalling in AOE-treated tumours was also consistent with reduced expression of several ERK signalling related genes, as identified by GO analysis of the microarray data. Indeed, significant down-regulation of *MAPK3, MAP2K2, MAPKAPK3, ELK1* and up-regulation of the phosphatase *DUSP1* was found in the reprogrammed tumours (Figure 4C). We next investigated whether the reduced activation of protein kinase signalling occurred immediately after the reprogramming or as long-term consequence of the extract treatment in tumour xenografts. We detected a reduction in expression of the early response genes *JUN*, but not *FOS* (Figure 4D) and an inhibition of p44/42 MAPK and p38MAPK after 6 hours reprogramming, but no change was found in JNK signalling (Figure 4E). Therefore, these data indicate inhibition of mitogen activated kinase signalling is an immediate consequence of the reprogramming process that is stably maintained in reverted tumours. Consistent with our findings, reverted tumours showed upregulated expression of *MAPK9* expression (Figure 4C), a negative regulator of JUN [24].

### Reprogramming with oocyte molecules induces a state of quiescence in cancer cells

**Figure 5:**
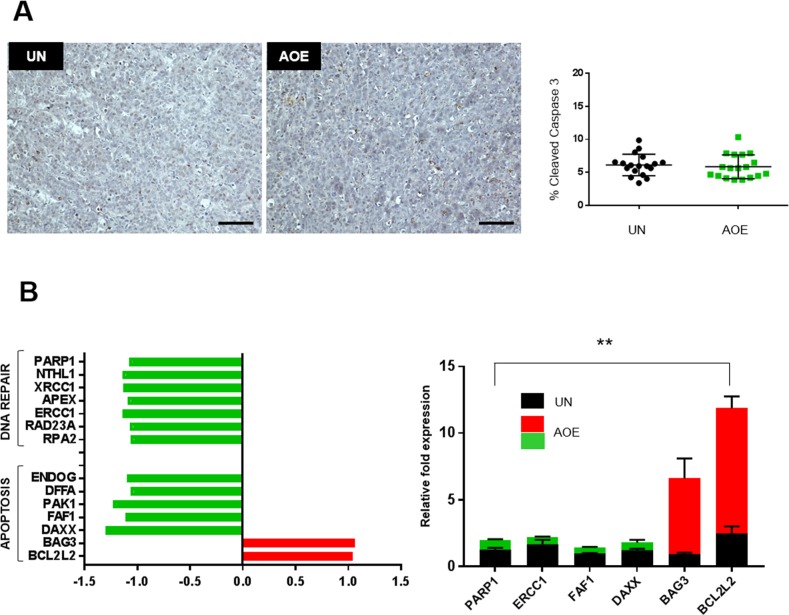
Quantification of apoptosis and DNA repair pathways **(A)** Cleaved Caspase 3staining of UN and AOE-treated tumour xenografts. Scatter plot shows the % of positively stained nuclei (n= 3; 6 fields of view). Staining levels were analysed by nonparametric Mann-Whitney test. p=0.4197. Scale bar = 100μm. **(B)** Fold change in apoptosis and DNA repair genes expression as determined by microarray analysis (left) and TaqMan^®^ qRT-PCR (right). Quantitative RT-PCR results for each gene are presented as relative fold expression to RPLP0 and the UN group used as calibrator (n=3). Relative fold expression levels were analysed by unpaired Student’s *t*-test. ^**^p< 0.01. Green and red bars indicate downregulated or upregulated genes in AOE, respectively.

We next asked whether the growth/proliferation arrest in reprogrammed tumours was associated with increased programmed cell death. Reprogrammed xenografts did not show any increase in caspase 3 activation (Figure 5A). Upregulation of the anti-apoptotic genes *BCL2L2, BAG3,* and downregulation of pro-apoptotic genes *DAXX, FAF1, PAK1, DFFA, ENDOG* was found in reprogrammed tumours pointing to a cell cycle arrest without engagement of the apoptotic pathway (Figure 5B). DNA damage response and repair pathways were also downregulated in reprogrammed tumours (Figure 5B).

**Figure 6:**
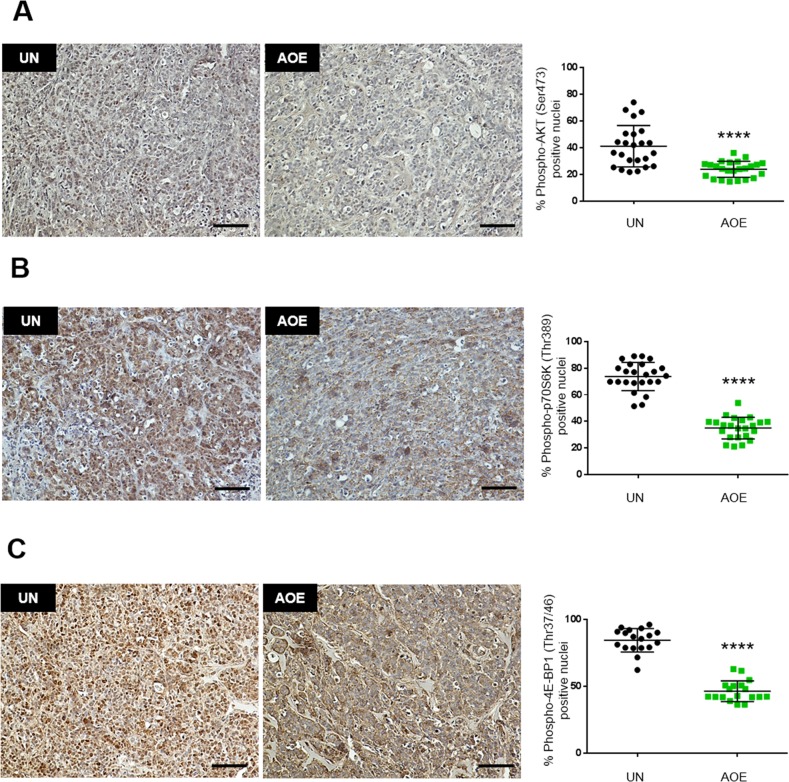
Quantification of AKT, p70S6K and 4E-BP1 activation in AOE-reprogrammed tumours **(A)** AKT phosphorylation staining of UN and AOE-treated tumour xenografts. Scatter plot shows the % of positively stained nuclei (n= 4; 6 fields of view). Staining levels were analysed by nonparametric Mann-Whitney test. ^****^p<0.0001. Scale bar = 100μm. **(B)** p70S6K phosphorylation staining of UN and AOE-treated tumour xenografts. Scatter plot shows the % of positively stained nuclei (n= 4; 6 fields of view). Staining levels were analysed by nonparametric Mann-Whitney test. ^****^p<0.0001. Scale bar = 100μm. **(C)** 4E-BP1 phosphorylation staining of UN and AOE-treated tumour xenografts. Scatter plot shows the % of positively stained nuclei (n= 3; 6 fields of view). Staining levels were analysed by nonparametric Mann-Whitney test. ^****^p<0.0001. Scale bar = 100μm.

To ascertain whether the growth-arrested tumours sustained long-term survival in a quiescent or senescent state, we next investigated signalling pathways that coordinate metabolic homeostasis in dormant cells. AOE-treated tumours showed reduced phosphorylation of AKT (Figure 6A) and reduced phosphorylation of both p70S6 kinase and the translation repressor 4E-BP1 (Figure 6B, 6C). Consistent with this, inhibition of protein synthesis was highlighted by GO analysis, where translation was found among the most significant biological processes in the down-regulated genes and EIF2 signalling was the top canonical pathway with predicted inhibition and a negative z-score (Supplementary Table 1).

Altogether, these results suggested a state of tumour dormancy. This could also explain the negative regulation of cellular metabolism found in the reprogrammed tumours, such as downregulated expression of genes involved in glucose metabolism (*TPI1, ENO1, ENO2, GALM, GPI, GAPDH, PFKP, PGM1, PDHB, PKM2*), oxidative phosphorylation (*ATP5D, UQCRC1, NDUFB9, NDUFV1, NDUFS8*) and DNA metabolism (*NANS, UGDH, AMDHD2, MPI, TK1*) in AOE-treated tumours. An association of these biological functions with the downregulated genes was also identified by GO and IPA analyses (Figure 1B, Supplementary Figure 1 and Supplementary Table 1).

**Figure 7:**
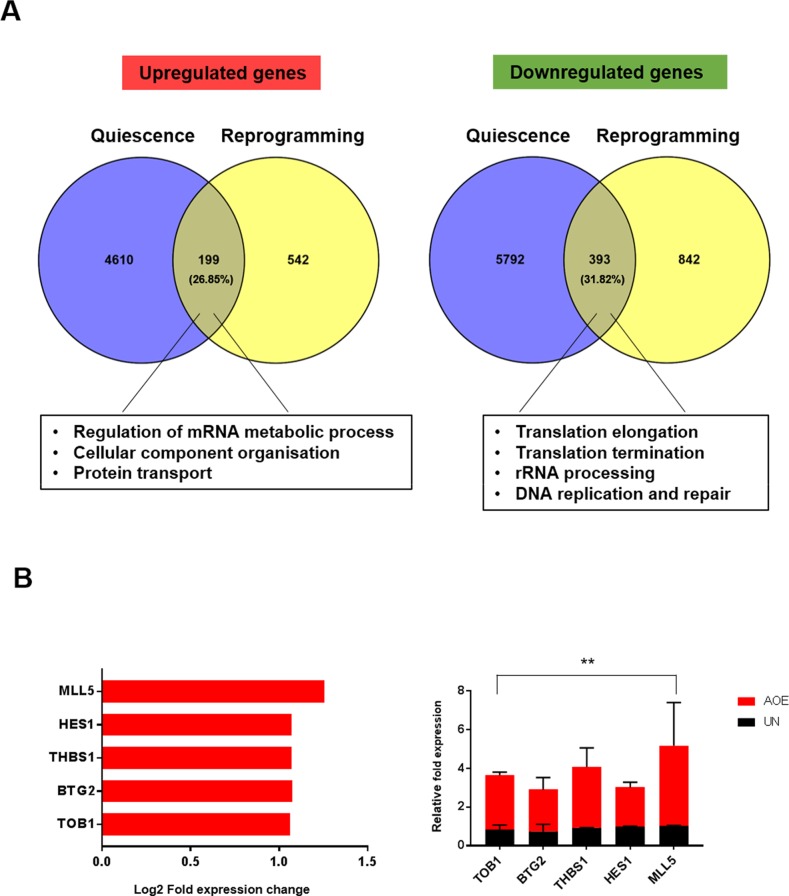
Gene expression signature of quiescence in AOE-reprogrammed tumour xenografts **(A)** Meta-analysis of gene expression profile of quiescent fibroblasts [25] and differentially expressed genes of AOE-reprogrammed tumours using Venny software. Enriched biological processes for overlapping upregulated and downregulated genes are highlighted. **(B)** Fold change in expression of quiescence-related gene in AOE-reprogrammed tumours as determined by microarray analysis (left) and TaqMan^®^ qRT-PCR (right). Quantitative RT-PCR results for each gene are presented as relative fold expression to *RPLP0* and the UN group used as calibrator (n=3). Relative fold expression levels were analysed by unpaired Student’s *t*-test. ^**^p< 0.01. Red bars indicate upregulated genes in AOE.

Because our data show that the dormant state induced by reprogramming is not associated with a response to oncogenic stress and increased mTOR activity that are characteristics of senescence, we next tested whether the lack of cell proliferation in reprogrammed tumours could be due to quiescence. Meta-analysis of data defining quiescence signatures in cell-cycle arrested fibroblasts [25] showed a substantial overlap (~30%) with the genes differentially expressed in reprogrammed tumours (Figure 7A). GO analysis showed that regulation of mRNA metabolic process, cellular component organisation and protein transport were among the most significant biological processes for the overlapping up-regulated genes, whereas translation elongation, translation termination, rRNA processing, DNA replication and repair were the most significant overlapping genes in the down-regulated group of genes. Importantly, the key regulators of quiescence TOB1, BTG2, THBS1, HES1 and MLL5 were found in the overlapping genes that were upregulated (Figure 7B). Thrombospondin-1 (THBS) is an anti-angiogenic factor and endothelium-derived tumour suppressor that can sustain breast cancer dormancy [26]. BTG proteins are important regulators of cell cycle progression [27] and TOB1 acts as a tumour suppressor in MCF-7 cells by inducing arrest at G1-S phase through upregulation of P27 and decreased activity of ERK2 and AKT [28].

### Oocyte extract mediated tumour quiescence involves epigenetic reprogramming

**Figure 8:**
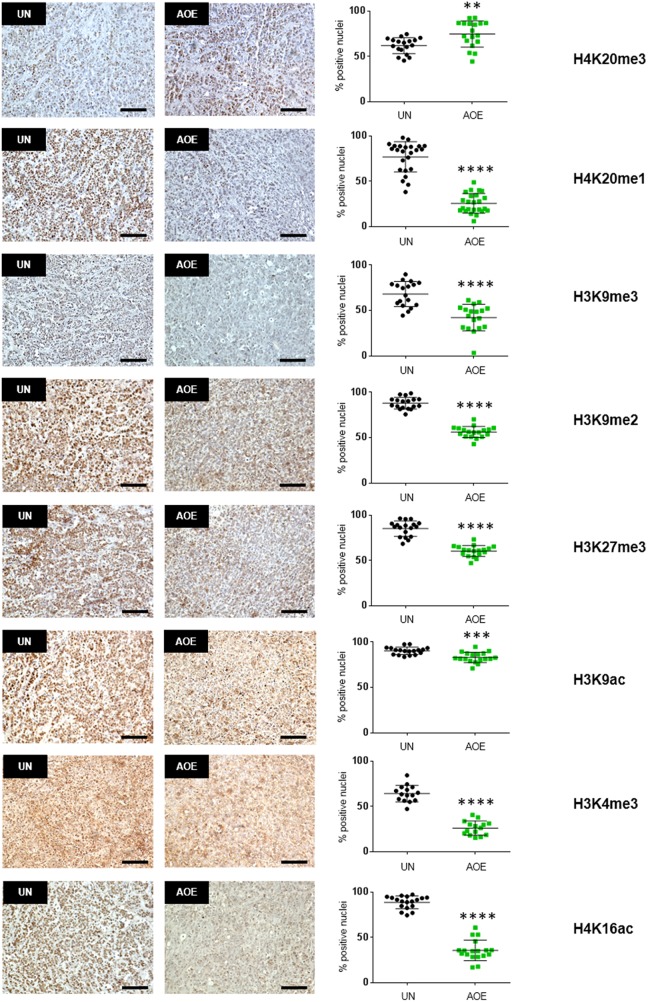
Quantification of histone modifications in AOE-reprogrammed tumours H4K20me3, H4K20me1, H3K9me3, H3K9me2, H3K27me3, H3K9ac, H3K4me3, H4K16ac staining of UN and AOE-treated tumour xenografts. Scatter plot shows the % of positively stained nuclei (n= 3-4; 6 fields of view). Staining levels were analysed by nonparametric Mann-Whitney test. ^**^p<0.01, ^***^p<0.001, ^****^p<0.0001. Scale bar = 100μm.

**Figure 9:**
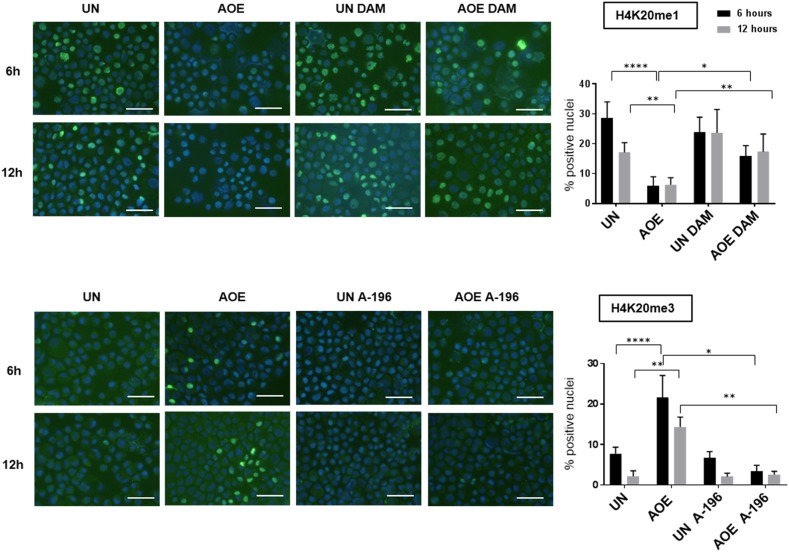
H4K20me1 and H4K20me3 staining of untreated and AOE-treated cells in the presence of inhibitors of SUV420H1/H2 and PHF8 H4K20me1 and H4K20me3 staining of cells incubated in growth medium (UN) or AOE for 6 hours in the presence or absence of Daminozide (DAM) or A-196. Cells were analysed immediately after reprogramming (6 hours) and after overnight culture (12 hours). Histogram shows the % of positively stained nuclei (n= 2; 5 fields of view). Staining levels were analysed by Two-way Anova followed by Bonferroni’s multiple comparisons test. ^*^p< 0.05. ^**^p<0.01, ^****^p<0.0001. Scale bar = 50μm.

We next investigated whether the induction of a quiescent state in treated tumours depended on epigenetic reprogramming, as previous work demonstrated the ability of AOE to remodel epigenetic modifications of the chromatin in both somatic and cancer cells [20, 29]. We therefore first analysed H4K20 histone methylation as this modification has been reported to regulate quiescence and chromatin compaction [30]. Consistent with an induction of quiescence, a significant decrease in the levels of H4K20me1 and increase in H4K20me3 were detected in reprogrammed tumours (Figure 8). In addition, similarly to the epigenetic profile reported in MCF-7 slowly proliferating “G0-like” cells [31], a decrease in H3K9me3, H3K9me2, H3K27me3 and H3K4me3 was found. Levels of active transcription marks H3K9ac and H4K16ac were also lower in reprogrammed tumours, confirming a non-cycling chromatin configuration (Figure 8). In order to investigate the relationship between epigenetic reprogramming and cell cycle arrest in the induction of quiescence we next studied the dynamics of H4K20 methylation and RB phosphorylation immediately after reprogramming. We measured a reduction of H4K20me1 and H4K20me3 after 6 hours and 12 hours of AOE treatment, in agreement with the epigenetic status of reprogrammed tumour xenografts. These changes were abolished when cells were incubated in extracts pre-treated and supplemented with the PHF8 inhibitor Daminozide (DAM) and the SUV420H1/H2 inhibitor A-196, which can prevent the demethylation and methylation of H4K20me1, respectively (Figure 9).

**Figure 10:**
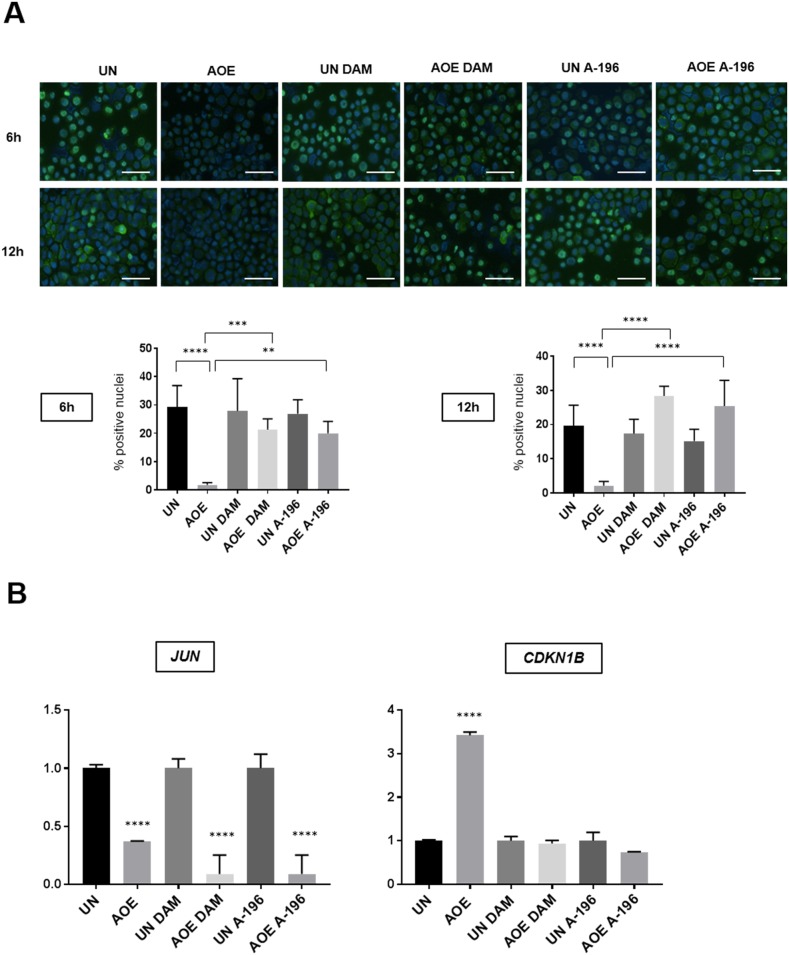
RB (Ser780) phosphorylation and expression of *JUN* and *CDKN1B (P27)* of untreated and AOE-treated cells in the presence of inhibitors of SUV420H1/H2 and PHF8 **(A)** RB (Ser780) phosphorylation staining of cells incubated in growth medium (UN) or AOE for 6 hours in the presence or absence of DAM or A-196. Cells were analysed immediately after reprogramming (6 hours) and after overnight culture (12 hours). Histogram shows the % of positively stained nuclei (n= 2; 5 fields of view). Staining levels were analysed by One-way Anova followed by Bonferroni’s multiple comparisons test. ^**^p<0.01, ^***^p<0.001, ^****^p<0.0001. Scale bar = 50μm. **(B)** Fold change in expression of *JUN* (6 hours after reprogramming) and *P27 (CDKN1B)* (12 hours after reprogramming) as determined by TaqMan^®^ qRT-PCR. Results are presented as relative fold expression to *RPLP0* and the UN group used as calibrator (n=3). Relative fold expression levels were analysed by One-way Anova followed by Bonferroni’s multiple comparisons test. ^****^p< 0.0001.

**Figure 11:**
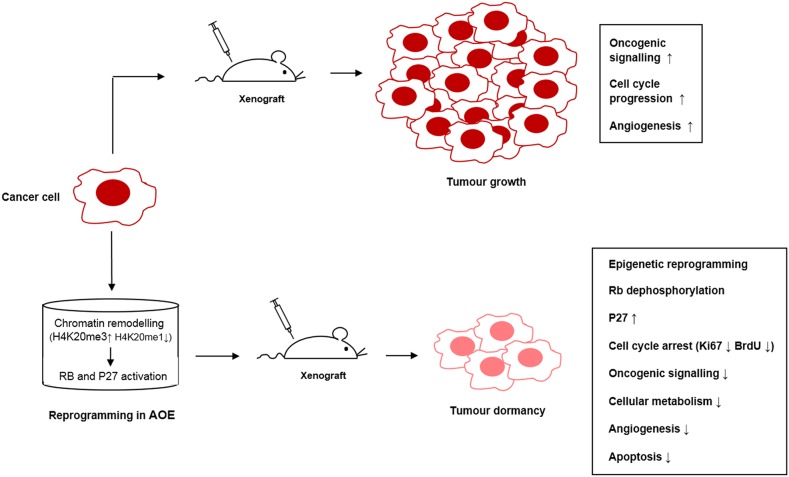
Pathways crosstalk involved in tumour reversion mediated by oocyte extracts

This inhibition was most likely due to an effect on histone demethylase and methylase enzymes contained in the extract as no effect was seen when inhibitors were added to control cells incubated in buffer under the same experimental conditions. Importantly, the AOE-mediated de-phosphorylation of RB did not occur in the presence of these epigenetic inhibitors (Figure 10A). Induction of CDKN1B (P27) expression also was dependent of H4K20me remodelling, whereas the reduced expression of *JUN* was not affected (Figure 10B). Therefore, the results suggest that the induction of a stable cell cycle arrest and associated tumour cellular dormancy depends on epigenetic reprogramming mediated by oocyte molecules which promote chromatin compaction and inhibition of the cell cycle (Figure 11).

## DISCUSSION

The idea of inducing tumour reversion by allowing cancer cells to regain control of normal growth has been receiving renewed attention [32]. Tumour reversion is considered a different approach for the development of new treatments based on the selection of stable cancer revertants. As a therapeutic strategy, tumour reversion can induce tumour differentiation or growth arrest by reactivating the normal control mechanisms that regulate cell proliferation. In this way, successful tumour reversion strategies can allow complete or partial stable cancer remission. Tumour reversion has also been an important strategy for elucidating of molecular mechanisms involved in tumorigenesis. For instance, tumour reversion induced by oncolytic viruses or viral oncogenes has led to the discovery of novel tumour suppressor genes [4, 33–35].

We previously showed that treatment of breast cancer cells with axolotl oocyte extracts can induce growth arrest both *in vitro* and *in vivo* [20]. In this study, we sought to identify the molecular mechanisms involved in such a growth-arrested phenotype to elucidate whether treatment of cancer cells with oocyte extracts could provide insight into potential therapeutic targets for tumour reversion. By genome-wide screening of reverted xenograft tumours we demonstrated that treatment of luminal breast cancer cells with oocyte extracts induces cell cycle arrest associated with upregulated expression of *CDKN1B* (*P27*) and reduction of RB phosphorylation. *CDKN1B* is a cell cycle inhibitor that controls the G0 to S- phase transition by regulating activity of the cyclin-dependent kinase (CDK) complexes CyclinD-CDK4 and CyclinD-CDK6 [36]. Decreased expression of P27 has been correlated with the development of and poor prognosis of breast cancer [37, 38]. Importantly, the oocyte extract treatment not only increased the expression of P27 but also changed its localisation from the cell cytoplasm to the nucleus. In quiescent normal cells, functional P27 has a nuclear localisation, whereas oncogenic activation of MAPK promotes its cytoplasmic accumulation, thus promoting cell motility and invasion [36]. Therefore, restoration of P27 expression and function represents a significant effect of oocyte-mediated tumour reversion. Consistent with inhibition of the G0/G1-S transition, we also measured reduced expression of CDK4 in reprogrammed tumours and reduced RB phosphorylation at Ser780 (the RB amino acid substrate of the Cyclin D1-CDK complex) in reprogrammed tumours [39]. In its active form, hypophosphorylated RB leads to repression of E2F-regulated genes and inhibition of progression through S phase and G2/M [40]. Reprogrammed tumours also presented reduced phosphorylation of p44/42, a pathway implicated in breast cancer growth. Indeed, mutations leading to constitutive activation of the p44/42 MAPK pathway is common in breast cancer and critical for breast cancer progression and invasion [41].

This pathway has been shown to mediate P27 degradation [42]. In turn, P27 can inhibit MAPK activation by sequestering H-Ras [43], suggesting that these mechanisms could reduce cell proliferation working through a negative feedback loop in AOE treated tumours.

JNK was another downregulated signalling pathway in growth arrested AOE-treated tumours. Activation of JNK has a suppressive role in mammary carcinogenesis by mediating stress-induced apoptosis [44, 45]. One unexpected finding in this study was the lack of apoptosis following cell cycle arrest induced by AOE treatment, which could be due to the inhibition of the observed JNK signalling. Suppression of cancer growth is not always associated with an apoptotic response, but it can also involve an induction of cellular dormancy, a state that can consist of either quiescence or senescence. In contrast to senescence, which occurs irreversibly in response to oncogenic signals and ageing, quiescence is a reversible process which is not induced by replicative stress. During quiescence cells become G0-G1 arrested due to lack of growth factor stimulation, cell adhesion signalling and angiogenesis, thus resulting in inhibition of metabolic and translational activity [46]. AOE-reprogrammed tumours also showed reduced activation of the AKT and mTOR pathways, which pointed to a quiescence phenotype, rather than senescence. Because quiescent cells demonstrate a characteristic gene expression profile which is not a mere consequence of cell cycle arrest [47], we overlapped our gene set with the quiescence-specific gene expression profile of quiescent fibroblasts [25]. The fact that we found a significant overlap between gene signatures confirms an induction of tumour dormancy after AOE reprogramming. Two important epigenetic regulators, MLL5 and HES1 [48, 49], were identified among the common quiescence-related genes, suggesting a link between AOE-induced growth arrest and epigenetic reprogramming. We previously demonstrated that AOE can induce profound epigenetic changes both in somatic and cancer cells which involve remodelling of DNA methylation and histone modifications [20, 50]. Therefore, one important question to address was the relationship between the cell cycle arrest and epigenetic reprogramming mediated by AOE. We found that AOE reprogrammed tumours demonstrated an extensive epigenetic reprogramming which involved the acquisition of a compact chromatin configuration [30]. Loss of H4K20me3 is a hallmark of cancer and a prognostic factors in breast cancer [51, 52]. Therefore, restoration of this chromatin mark is likely to be an important regulator of AOE-mediated tumour reversion. We have shown that epigenetic reprogramming is initiated during the 6 hours treatment of cancer cells with AOE and is maintained up to the point when cells are injected into mice. However, this reprogramming was halted when inhibitors of epigenetic modifiers were used. In addition, the inhibition of the these enzymatic activities prevented RB dephosphorylation and induction of P27 expression, suggesting that epigenetic reprogramming due to chromatin compaction may be critical for cell cycle arrest, as previously reported [53]. Because the inhibitors did not affect cells that were not treated with AOE (control group), we speculated that the epigenetic reprogramming may be mediated by enzymatic activities present in the oocyte extracts. Mammalian oocytes express H4K20me modifying enzymes [54] and oocyte-specific factors can dynamically contribute to the reprogramming process by assimilation into reprogrammed cells [15, 55]. In addition, un-ovulated oocytes are arrested in prophase of meiosis I through inhibition of p44/42 MAPK phosphorylation [56]. Therefore, it is possible that oocyte molecules that are naturally involved in the maintenance of oocyte cell cycle arrest could be assimilation into cancer cells and mediate tumour reversion.

Induction of primary tumour dormancy represents an attractive therapeutic strategy for cancer treatment [57, 58]. Our data show that AOE reprogramming of cancer cells to a quiescent state is initiated early during the extract treatment and maintained stably during the development of xenografts over time (at least up to 15 months, data not shown). Therefore, oocyte extracts may contain molecules involved in inducing stable chemoquiescence and tumour reversion. Although this study is limited to the analysis of tumour reversion in luminal breast cancer cells, it highlights the importance of tumour reversion approaches based on cell cycle arrest and activation of the RB pathway. RB signalling plays a critical role in breast cancer and therefore therapeutic strategies that harness this pathway have a potential for clinical intervention [59]. This is particularly relevant for luminal-type breast cancer whereby reactivation of the RB pathway is beneficial for the treatment of tumours resistant to endocrine therapies [60]. Therefore, together with CDK inhibitors, purified molecules from axolotl oocytes could contribute to the development of new dormancy-promoting therapies aimed to maintain tumours in an asymptomatic state as well as to strategies for cancer prevention.

## MATERIALS AND METHODS

All cell culture materials were from Invitrogen and chemicals from Sigma-Aldrich, unless otherwise stated.

### Cell culture and extract treatment

The breast cancer cell line MCF-7 was obtained from ATCC and was grown in RPMI-1640 medium supplemented with 10% foetal calf serum (FCS), 1% Penicillin/Streptomycin (Pen/Strep), 1% L-Glutamine, 1% sodium pyruvate, 1% non-essential amino acids. Cell line identity was validated by genotyping (Bio-Synthesis Inc) and cells were regularly checked for mycoplasma contamination using the EZ-PCR Mycoplasma Test Kit (Geneflow Ltd). For extract treatment, cell permeabilisation was performed as previously reported [20, 55]. Briefly, cell suspensions (2×10^6^ cells/ml) were treated with 20 μg/ml digitonin in PB buffer (170 mM potassium gluconate, 5 mM KCl, 2 mM MgCl2, 1 mM KH2PO4, 1 mM EGTA, 20 mM Hepes, supplemented with 1:5,000 dilution of Protease Inhibitor Cocktail, pH 7.25) for 1-2 min on ice. Cells were washed in cold PB buffer and permeabilisation was assessed by staining with propidium iodide (PI). AOE were prepared from mature females as described previously [55]. Permeabilised cells were added either to growth medium (untreated group) or oocyte extracts (5,000 cells/μl extract) supplemented with an energy regenerating system (150 μg/ml creatine phosphokinase, 60 mM phosphocreatine, 1mM ATP) and incubated at 17°C. After reprogramming, cells were plated in growth medium overnight. On the next day, cells were collected and viability assessed by trypan blue stain. Only cultures with more than 95% viability were used for injection into immunocompromised mice or other characterisation studies. For epigenetics studies, AOE was pre-treated with 10 μM Daminozide (DAM) (Tocris Bioscience) or 10 μM A-196 (kindly provided by SGC, www.thesgc.org) 2 hours at 17°C. The inhibitors were maintained in both AOE and buffer control during the 6 hours reprogramming. After this, cells were washed and incubated in culture medium for analysis at 12 hours.

### Tumour xenografts

Female MF1 nude mice (Harlan-Olac) were injected sub-cutaneously into the left flank with 1.5×106 MCF-7 cells (untreated and AOE-treated) in a volume of 200μl Matrigel. 17-beta-estradiol pellets (0.1mg, 60-day release; Innovative Research of America, US) were implanted subcutaneously into the scruff of each mouse (n=6). Tumour dimensions were monitored twice weekly by calliper measurements. For BrdU incorporation experiments, BrdU (10 mg/ml) was injected at a dose of 150mg/kg via intraperitoneal injection 60 minutes before sacrifice. At termination (11 weeks), tumours were excised, stored in RNAlater (Thermo Scientific) or fixed in formalin and paraffin embedded. The project was run under Home Office project PPL 40/2962 with local ethical approval. The study adhered to the UK Co-ordinating Committee for Cancer Research (UKCCCR) guidelines.

### Immunohistochemistry

Histology sections (5 μm) were de-waxed and processed for antigen retrieval (citrate buffer pH 6, 15 min using microwave). Slides were washed with PBS and treated with Bloxall blocking solution (Vector Laboratories). Slides were then incubated with Mouse on Mouse blocking reagent (Vector Laboratories) and 2.5% normal horse serum (1 hour, RT) before incubation with primary antibody at 4°C overnight. This was then followed washes with PBS containing 0.1% Tween 20 and by incubation with secondary antibody for 1 hour at RT (ImmPRESS kit, Vector laboratories). After further washing, the slides were incubated with DAB for 1-5 minutes, counterstained with haematoxylin, dehydrated and mounted with DPX. For BrdU staining, sections were treated with PBS containing 0.25% Triton X-100 and 0.1% goat serum for 10 min at room temperature (RT) and blocked with PBS containing 10% goat serum for 1h at RT. After blocking, sections were incubated with 0.02U/ul DNAse in DNAse buffer (Qiagen) for 10 min at 37°C and then washed with PBS 3 times for 10 min at RT before incubation with the primary antibody. Tissue section slides were observed under a Nikon Eclipse microscope with the Nikon NIS-Elements imaging software.

For cell staining, slides were prepared using cytospin. After centrifugation, cells were fixed with 4% paraformaldehyde and then permeabilised with PBS containing 0.1% Triton X-100 for 10 minutes at RT. Slides were blocked with PBS containing 5% bovine serum albumin (BSA) for 1 hour at RT, followed by incubation with primary antibody overnight at 4°C and secondary antibody 1 hour at RT. Slides were washed after each antibody with PBS containing 0.1% Tween 20 and mounted with Vectashield mounting medium containing DAPI (Vector Laboratories). Slides were observed under a Leica DM5000B microscope with the Leica Application Suite software. For image quantification, 3-4 tumours were analysed for each treatment group and 5-6 random fields of view were analysed with the software Fiji-ImageJ. Quantification of images was performed by calculating the % of positive cells based on the ratio between the area stained by antibodies and the area of counterstained nuclei. Antibodies used are listed in Supplementary Table 2.

### Gene expression analysis

Total RNA was extracted with the RNeasy Mini Kit (Qiagen) and cDNA transcribed with the RT2 First Strand Kit (Qiagen). Real-time PCR (qRT-PCR) data analysis was performed by using TaqMan^®^ gene expression assays and master mix (Applied Biosciences). Assays used are listed in Supplementary Table 2. Data analysis was performed by using the relative quantification ΔΔCT method with normalization to the housekeeping genes RPLP0 which was selected after analysis of stability across samples with the software BestKeeper [61]. Gene expression of tumour xenografts (two biological replicates in technical triplicates) was analysed by microarray hybridisation using the Illumina HumanHT-12 v4 Expression BeadChip array (Source BioScience). The concentration and quality of the total RNA was assessed by spectrophotometry and using the Agilent Bioanalyser. Samples were normalised to 100ng and were processed according to the Illumina Whole-Genome Gene Expression Direct Hybridisation Assay Guide, using the Ambion Kit: Illumina^®^ TotalPrep™-96 RNA Amplification Kit. Qualitative & quantitative QCs were performed on the labelled cRNA and 1.5ug of labelled cRNA was hybridised to a HumanHT-12.v4 Beadchip & scanned by the BeadArray Reader. The array intensity data was analysed by the Illumina GenomeStudio software v2010.2. All analyses reported used the ‘quantile’ normalisation method with background correction within GenomeStudio.

Bioinformatics analysis of differentially expressed genes was performed using the ArrayMining software [62] with the Empirical Bayes moderated *t*-test method. Significant difference in expression was determined by FDR corrected p-value from multiple *t*-tests < 0.05. Microarray data were deposited in GEO with accession number GSE104383.

Gene ontology analyses were performed using the programs AmiGO 2 [63] and REVIGO [64]. The gene networks, pathways and functional analyses were generated through the use of QIAGEN’s Ingenuity Pathway Analysis (IPA^®^, QIAGEN Redwood City, www.qiagen.com/ingenuity) [65].

Meta-analysis of published data was performed by using the software Venny 2.1.0 (http://bioinfogp.cnb.csic.es/tools/venny/).

### Western blotting

Proteins were extracted with RIPA Buffer (Cell Signalling Technology), supplemented with Protease and Phosphatase Inhibitor Cocktails for 30 minutes on ice. Cells were centrifuged at 12,000 x g for 10 minutes at 4°C and protein lysate collected. The protein content was quantified using a Qubit Protein Assay Kit (Life Technologies) following the manufacturer’s protocol. Proteins were loaded into 8-12% polyacrylamide gels (30 μg/lane), separated by SDS-PAGE electrophoresis and blotted onto a PVDF membrane. Membranes were blocked with 5% BSA and then probed overnight at 4°C with primary antibodies. Peroxidase conjugated secondary antibodies were incubated for 1h at RT. ECL Prime (GE Healthcare) was used to detect chemiluminescence. Membranes were stripped with 0.2M glycine, pH 2.8 at 50°C for 30 min followed by neutralisation with 0.2M glycine, pH 7.4. Membranes were then blocked and re-probed with a control antibody. Antibodies used are listed in Supplementary Table 2.

### Statistics

Data are expressed as mean ± standard deviation (SD) unless otherwise stated. Quantitative RT-PCR analyses were analysed by Student’s *t*-test and One-way or Two-way analysis of variance (Anova) followed by Bonferroni’s multiple comparison test. Immunostaining data were analysed by Mann-Whitney test and One/Two-way Anova followed by Bonferroni’s multiple comparison test. Statistical analyses were performed with GraphPad Prism 7 with significance levels set at ^*^p< 0.05, ^**^p<0.01, ^***^p<0.001, ^****^p<0.0001.

## SUPPLEMENTARY MATERIALS FIGURES AND TABLES







## Abbreviations

AOE: axolotl oocyte extracts
UN: untreated
GO: gene ontology
IPA: ingenuity pathway analysis
mRNA: messenger RNA
rRNA: ribosomal RNA
MAPK: mitogen activated protein kinase
SAPK/JNK: stress activated protein kinase/c-jun N-terminal kinase
AKT: protein kinase B
mTOR: mechanistic target of rapamycin
ERK: extracellular signal regulated kinase
CDK: cyclin dependent kinase
RB: retinoblastoma protein
CDKN1B: cyclin dependent kinase inhibitor 1B
EIF2: eukaryotic translation initiation factor 2
TOB1: transducer of ERBB2, 1
BTG2: BTG anti-proliferation factor 2
THBS1: thrombospondin 1
HES1: Hes family BHLH transcription factor 1
MLL5: lysine methyltransferase 2E
JUN: Jun proto-oncogene, AP-1 transcription factor subunit
FOS: Fos proto-oncogene, AP-1 transcription factor subunit
RPLP0: ribosomal protein lateral stalk subunit P0
PHF8: Jumonji C domain-containing histone demethylase 1F
SUV420H1/H2: lysine methyltransferase 5B/5C
DAM: daminozide
FCS: fetal calf serum
PBS: phosphate buffered saline
PB: permeabilisation buffer
PI: propidium iodide
BrdU: bromodeoxyuridine
RT: room temperature
BSA: bovine serum albumin
FDR: false discovery rate
SD: standard deviation
qRT-PCR: real time quantitative PCR
GEO: gene expression omnibus
