# Supplementary material for: Cancer reversion with oocyte extracts is mediated by cell cycle arrest and induction of tumour dormancy
Source: Oncotarget. 2018 Mar 23;9(22):16008–27. doi: 10.18632/oncotarget.24664 (PMC5882314; doi:10.18632/oncotarget.24664)
Supplement: Supplementary file 1 [file oncotarget-09-16008-s001.pdf]

Cancer reversion with oocyte extracts is mediated by cell cycle arrest and induction of tumour dormancy

SUPPLEMENTARY MATERIALS

A

TOP NETWORKS (Associated network functions)	Score
Developmental Disorder, Hereditary disorder, Metabolic disease	44
Post-translational modification, Protein folding, Cellular assembly and organisation	44
Hereditary disorder, Metabolic disease, Lipid metabolism	44
DNA replication, recombination and repair, Hereditary disorder, Neurological disorder	41
Cell death and survival, cellular growth and proliferation, Cell-to-cell signalling and interaction	41

TOP BIO FUNCTIONS (Molecular and Cellular functions)	P value	Molecules
Protein synthesis	1.17E-02-7.11E-11	197
RNA post-transcriptional modification	9.30E-03-2.05E-10	67
Gene expression	1.49E-02-5.53E-10	315
Cellular growth and proliferation	1.49E-02-7.39E-09	463
Cell death and survival	1.49E-02-7.50E-09	463

B

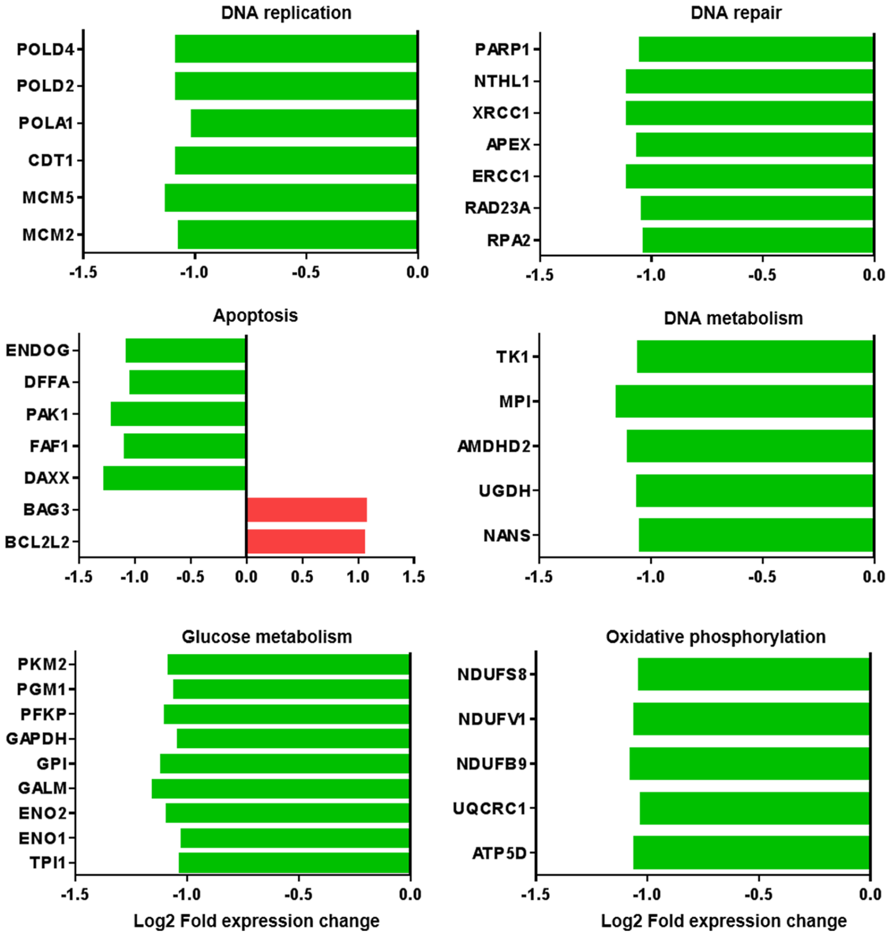

Supplementary Figure 1: Gene expression profile of AOE-treated tumour xenografts. (A) Top five Networks and Bio Functions identified by IPA. **(B)** Fold change in expression of genes involved in DNA replication, DNA metabolism, glucose metabolism and oxidative phosphorylation as determined by microarray analysis.

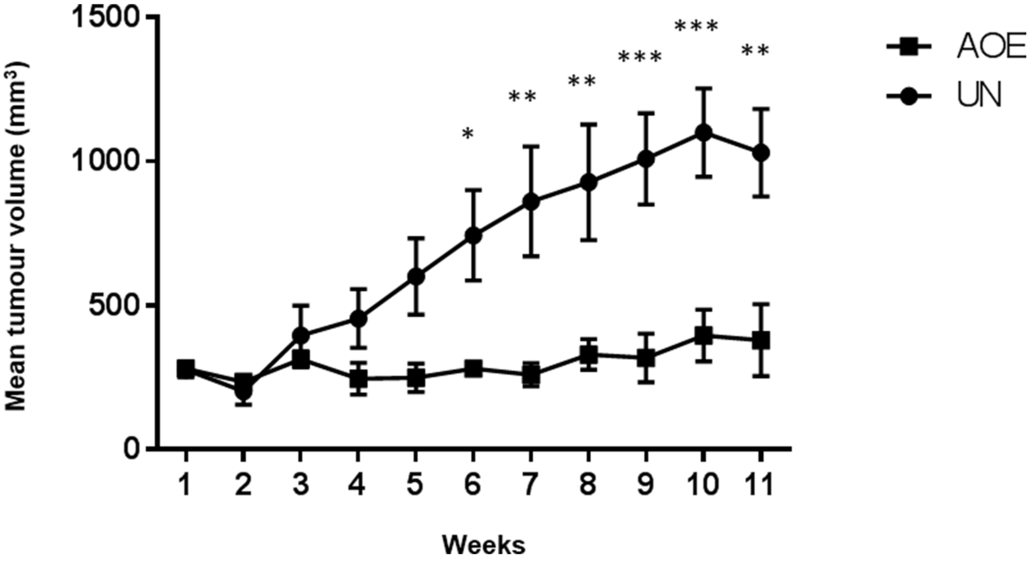

Supplementary Figure 2: Growth of AOE-treated tumour xenografts. Growth curve of tumour xenografts. MCF-7 cells (untreated and AOE-treated, 1.5×10^6) were injected sub-cutaneously with Matrigel into the left flank of female MF1 nude mice receiving 17-beta-estradiol pellets (n=6). Tumour dimensions were monitored twice weekly by calliper measurements. For BrdU incorporation experiments, BrdU (10 mg/ml) was injected at a dose of 150mg/kg via intraperitoneal injection 60 minutes before sacrifice. At termination (11 weeks), tumours were excised and analysed. Data were analysed by Two-way Anova followed by Bonferroni's multiple comparisons test. *p<0.05, **p<0.01, ***p<0.001.

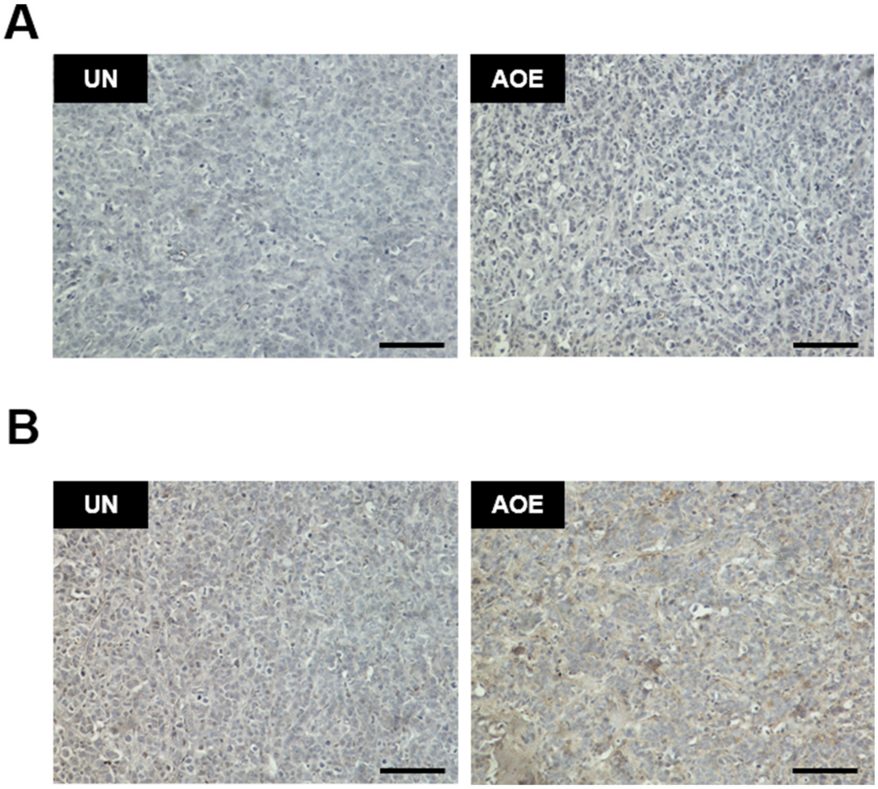

Supplementary Figure 3: Levels of phospho-RB (Ser795) and p38MAPK in AOE-reprogrammed tumours. (A) Phospho-RB (Ser795) staining of UN and AOE-treated tumour xenografts. Scale bar = 100μm. **(B)** p38MAPK staining of UN and AOE-treated tumour xenografts. Scale bar = 100μm.

Supplementary Table 1: Most significant canonical pathways.

See Supplementary File 1

Supplementary Table 2: PCR assays and antibodies used in this study.

See Supplementary File 2
