# Supplementary material for: Cancer reversion with oocyte extracts is mediated by cell cycle arrest and induction of tumour dormancy
Source: Oncotarget. 2018 Mar 23;9(22):16008–27. doi: 10.18632/oncotarget.24664 (PMC5882314; doi:10.18632/oncotarget.24664)
Supplement: Supplementary file 2 [file oncotarget-09-16008-s002.docx]

**Supplementary Table 1: Most significant canonical pathways.**

| **Ingenuity Canonical Pathways** | **-log**  **(p-value)** | **Ratio** | **Z-score** | **Molecules** |
| --- | --- | --- | --- | --- |
| EIF2 Signalling | 8.97E00 | 2.11E-01 | -2.065 | RAF1,RPL27A,RPS3A,RPS18,RPS23,  RPS11,RPS28,RPL14,MAP2K2,RPL18A,  RPS20,EIF3D,RPL23A,MAPK3,RPS9,EIF5,GSK3B,RPL36,RPS3,PPP1CA,RPL18,  RPL13,PABPC1,RPL4,NRAS,RPL3,RPS2,  EIF3J,RPL12,EIF3E,EIF3G,FAU,RPS16,  RPL5,EIF3L,RPL13A,EIF3K,RPS14,RPL38 |
| Mitochondrial Dysfunction | 5.75E00 | 1.81E-01 |  | HSD17B10,ATP5D,COX6C,RHOT2,TRAK1,CYB5R3,ATP5E,NDUFA2,NDUFB9,GPX4,  ATP5J,NDUFV1,COX17,CPT1A,UCP2,  SURF1,COX7C,MAPK9,APP,FIS1,  NDUFB11,ATP5B,NDUFS8,NDUFA11,  COX7A2,APH1B,BACE2,ATPAF2,UQCRC1UQCRQ,NDUFB2 |
| Germ Cell-Sertoli Cell Junction Signalling | 4.96E00 | 1.75E-01 |  | MAP3K11,RHOT2,BCAR1,PAK1,AGGF1,  RHOG,MAP2K2,RHOB,RHOT1,MAPK3,  RHOF,EPN1,NRAS,CFL1,TJP1,TUBB4B,  TNFRSF1A,TUBB2A,MAPK9,CDH1,TUBB6,ACTA2,RHOA,TGFB3,ZYX,ACTN4,CLINT1,PVRL2 |
| mTOR Signalling | 4.88E00 | 1.65E-01 | -0.577 | PRKAB1,RPS3A,RPS18,RHOT2,RPS23,  PRKAG1,RPS11,RPS28,RHOG,RPS20,  RHOB,RHOT1,EIF3D,MAPK3,RPS9,RHOF,RPS3,EIF4B,NRAS,RPS2,EIF3J,EIF3E,  MLST8,EIF3G,PPP2R1A,FAU,RPS16,  RHOA,EIF3L,EIF3K,RPS14 |
| Protein Ubiquitination Pathway | 4.41E00 | 1.45E-01 |  | CRYAB,HSPA14,PSMD7,HLA-A USP5,USP11,DNAJC12,HSPA1A/HSPA1B,DNAJA1,TCEB2,UBE4A,DNAJC4,DNAJC30DNAJC22,AMFR,PSMA6,UBB,DNAJC9,  HSPH1,PSMD5,THOP1,PSMD3,HSPA8,  PSMC1,DNAJC5,HSCB,PSMD2,DNAJB11,BAP1,HSP90AA1,PSMD1,CDC34,UBA1,  USP34,BIRC2,HSPB1,UBE2I |
| Regulation of eIF4 and p70S6K Signalling | 4.33E00 | 1.71E-01 | 0.447 | RAF1,RPS3A,EIF4EBP2,RPS18,RPS23,  RPS11,RPS28,RPS20,MAP2K2,EIF3D,  MAPK3,RPS9,RPS3,PABPC1,NRAS,RPS2,EIF3J,EIF3E,EIF3G,FAU,PPP2R1A,RPS16,EIF3L,RPS14,EIF3K |
| Breast Cancer Regulation by Stathmin1 | 4.33E00 | 1.57E-01 |  | RAF1,PPP1R3C,GNB2L1,E2F3,PRKAG1,  ROCK2,ARHGEF19,PAK1,MAP2K2,  PPP1R7,MAPK3,E2F5,RB1CC1,GNA13,  PPP1CA,CALML5,NRAS,TUBB4B,TUBB2A,ITPR1,GNG10,GNAI2,CALM1,ARHGEF5,  PPP2R1A,TUBB6,RHOA,CDKN1B,CDK2,  CAMK2G |
| Antiproliferative Role of TOB in T Cell Signalling | 4.27E00 | 3.46E-01 |  | PABPC1,CCNA2,PABPC4,TGFB3,TOB1,  CDC34,CDKN1B,TWSG1,CDK2 |
| Semaphorin Signalling in Neurons | 4.11E00 | 2.45E-01 |  | DPYSL2,CFL1,RHOT2,ROCK2,PAK1,  SEMA4D,RHOG,RHOB,RHOT1,MAPK3,  RHOA,RHOF,ARHGAP1 |
| Chronic Myeloid Leukemia Signalling | 3.98E00 | 1.94E-01 |  | RAF1,NRAS,TFDP1,SUV39H1,CDK4,  IKBKE,CRK,E2F3,SIN3A,CTBP1,BCL2L1,  MAP2K2,HDAC11,MAPK3,E2F5,TGFB3,  CHUK,CDKN1B |
| Integrin Signalling | 3.91E00 | 1.49E-01 | -1.512 | RAF1,MAP3K11,RHOT2,MYL5,CRK,  BCAR1,ITGAE,TSPAN3,PAK1,RHOG,  MAP2K2,RHOB,RHOT1,ARF4,MAPK3,  CAV1,ITGAV,GSK3B,RHOF,MYL12A,  ACTR2,NRAS,ASAP1,ITGB2,CAPNS1,  ARF5,ACTA2,RHOA,ZYX,ACTN4 |
| Fatty Acid β-oxidation I | 3.73E00 | 3E-01 |  | HSD17B10,ACSL3,ECHS1,SLC27A1,IVD,  ACADM,ECI1,HADHA,HADH |
| CXCR4 Signalling | 3.62E00 | 1.58E-01 | -0.655 | RAF1,NRAS,RHOT2,MYL5,GNB2L1,  MAPK9,CRK,ITPR1,BCAR1,GNG10,GNAI2,ROCK2,ELMO3,PAK1,RHOG,MAP2K2,  RHOB,RHOT1,MAPK3,RHOA,GNA13,  RHOF,ELK1,MYL12A |
| Aldosterone Signalling in Epithelial Cells | 3.62E00 | 1.58E-01 |  | RAF1,CRYAB,HSPA14,DNAJC9,HSPH1,  SLC12A2,HSPA1A/HSPA1B,DNAJC12,  ITPR1,DNAJA1,HSPA8,SCNN1A,DNAJC5,MAP2K2,DUSP1,DNAJC4,HSCB,PIP5K1C,MAPK3,DNAJB11,HSP90AA1,DNAJC30,  DNAJC22,HSPB1 |
| Oxidative Phosphorylation | 3.54E00 | 1.74E-01 |  | ATP5J,NDUFV1,COX17,ATP5D,COX6C,  COX7C,SURF1,NDUFA2,ATP5E,NDUFB9,NDUFB11,ATP5B,NDUFS8,NDUFA11,  COX7A2,ATPAF2,UQCRC1,UQCRQ,  NDUFB2 |
| tRNA Charging | 3.46E00 | 2.56E-01 |  | WARS2,WARS,TARS2,VARS2,DARS,  TARS,SARS2,SARS,FARSA,QARS |
| Cyclins and Cell Cycle Regulation | 3.38E00 | 1.92E-01 |  | RAF1,TFDP1,SUV39H1,WEE1,CDK4,E2F3,SIN3A,CCNA2,PPP2R1A,HDAC11,TGFB3,E2F5,GSK3B,CDKN1B,CDK2 |
| Cell Cycle: G1/S Checkpoint Regulation | 3.24E00 | 2.03E-01 | -0.632 | TFDP1,SUV39H1,CDK4,E2F3,SIN3A,  FOXO1,HDAC11,E2F5,TGFB3,RPL5,  CDKN1B,GSK3B,CDK2 |
| RhoGDI Signalling | 3.15E00 | 1.45E-01 | 1.528 | RHOT2,MYL5,GNB2L1,ROCK2,ARHGEF1,PAK1,RHOG,RHOB,RHOT1,CDH3,GNA13,RHOF,MYL12A,ACTR2,CFL1,WASF2,GDI2,GNG10,GNAI2,ARHGEF5,CDH1,ACTA2,  PIP5K1C,RHOA,ARHGAP1 |
| Glycogen Degradation III | 2.99E00 | 4.17E-01 |  | GAA,PGM5,PGM1,PYGB,PYGL |
| Sertoli Cell-Sertoli Cell Junction Signalling | 2.97E00 | 1.4E-01 |  | RAF1,MAP3K11,BCAR1,PRKAG1,AGGF1,MAP2K2,MAPK3,GSK3B,EPN1,NRAS,  TNFRSF1A,TJP1,TUBB4B,TUBB2A,MAPK,CDH1,TJP3,TUBB6,ACTA2,TGFB3,ACTN4,CLINT1,ELK1,CLDN3,PVRL2 |
| Estrogen-mediated S-phase Entry | 2.93E00 | 2.92E-01 | 0.000 | CCNA2,TFDP1,E2F5,CDK4,CDKN1B,E2F3,CDK2 |
| Glycolysis I | 2.81E00 | 2.8E-01 |  | ENO1,GPI,TPI1,PKM,ENO2,GAPDH,PFKP |
| Glucocorticoid Receptor Signalling | 2.81E00 | 1.24E-01 |  | RAF1,PRKAB1,HSPA14,HSPA1A/HSPA1B,SMARCD2,GTF2E2,POLR2B,TAF13,  PRKAG1,PTGES3,MAP2K2,MAPK3,  SMARCB1,CHUK,TAF2,CREBZF,POLR2I,  NRAS,MAPK9,IKBKE,ERCC2,HSPA8,  BCL2L1,TAF6L,POU2F1,TAF4,VIPR1,  DUSP1,TGFB3,HSP90AA1,NCOR2,ELK1,  PHF10,UBE2I |
| IL-8 Signalling | 2.77E00 | 1.36E-01 | 0.000 | RAF1,RHOT2,GNB2L1,ROCK2,RAB11FIP,RHOG,RHOB,MAP2K2,RHOT1,MAPK3,  ITGAV,GNA13,CHUK,RHOF,LASP1,NRAS,MAPK9,IKBKE,CSTB,GNG10,GNAI2,  BCL2L1,ITGB2,CDH1,RHOA |
| Colanic Acid Building Blocks Biosynthesis | 2.64E00 | 3.57E-01 |  | TSTA3,GPI,UGDH,GALK1,MPI |
| DNA Methylation and Transcriptional Repression Signalling | 2.64E00 | 3E-01 |  | CHD4,MBD3,RBBP7,DNMT1,SIN3A,RBBP4 |
| Cholecystokinin/Gastrin-mediated Signalling | 2.62E00 | 1.58E-01 | 0.000 | RAF1,NRAS,RHOT2,MAPK9,ITPR1,BCAR,ROCK2,RHOG,RHOB,MAP2K2,RHOT1,  RHOA,MAPK3,GNA13,RHOF,ELK1 |
| Cell Cycle Control of Chromosomal Replication | 2.6E00 | 2.59E-01 |  | MCM5,MCM6,MCM2,CDT1,CDK4,CDK2,  RPA2 |
