# Supplementary material for: Cancer reversion with oocyte extracts is mediated by cell cycle arrest and induction of tumour dormancy
Source: Oncotarget. 2018 Mar 23;9(22):16008–27. doi: 10.18632/oncotarget.24664 (PMC5882314; doi:10.18632/oncotarget.24664)
Supplement: Supplementary file 3 [file oncotarget-09-16008-s003.docx]

**Supplementary Table 2: PCR assays and antibodies used in this study.**

| **PCR assays** |  | |
| --- | --- | --- |
| **Gene** | **Assay ID** |  |
| *BTG2* | Hs00198887_m1 |  |
| *CDK4* | Hs00175935_m1 |  |
| *CDKN1B* | Hs01597588_m1 |  |
| *CDKN2B* | Hs00793225_m1 |  |
| *CDKN3* | Hs00193192_m1 |  |
| *E2F5* | Hs00231092_m1 |  |
| *FOS* | Hs04194186_s1 |  |
| *HES1* | Hs00172878_m1 |  |
| *JUN* | Hs99999141_s1 |  |
| *MLL5* | Hs01096121_m1 |  |
| *TGFB3* | Hs01086000_m1 |  |
| *THBS1* | Hs00962908_m1 |  |
| *TOB1* | Hs03986111_s1 |  |
| *WEE1* | Hs00268721_m1 |  |
| **Antibodies** |  | |
| **Antibody** | **Manufacturer** | **Antibody final concentration/dilution** |
| Mouse anti-BrdU | Roche, 11170376001 | 0.6 µg/ml |
| Mouse anti -Ki67 | eBioscience, 14-5699 | 0.1 µg/ml |
| Rabbit anti-P27 | Cell Signalling Technology, 3686 | 0.1 µg/ml |
| Rabbit anti-phospho RB (Ser780) | Cell Signalling Technology, 9307 | 1:200 |
| Rabbit anti-phospho RB (Ser807/811) | Cell Signalling Technology, 9308 | 1:300 |
| Rabbit anti-phospho RB (Ser795) | Cell Signalling Technology, 9301 | 1:200 |
| Mouse anti-RB | Cell Signalling Technology, 9309 | 1:100 |
| Rabbit anti-cleaved caspase 3 (Asp175) | Cell Signalling Technology, 9661 | 1:300 |
| Rabbit anti-phospho p44/42 MAPK | Cell Signalling Technology, 4370 | 1:400 (immunohistochemistry)  1: 2000 (western blotting) |
| Rabbit anti-phospho p38 MAPK | Cell Signalling Technology, 4511 | 1:400 (immunohistochemistry)  1: 1000 (western blotting) |
| Rabbit anti-phospho SAPK/JNK | Cell Signalling Technology, 4668 | 1:100 (immunohistochemistry)  1: 1000 (western blotting) |
| Rabbit anti-phospho AKT(Ser473) | Cell Signalling Technology, 4060 | 1:50 |
| Rabbit anti-phospho p70S6K(Thr389) | Cell Signalling Technology, 9234 | 1:200 |
| Rabbit anti-phospho 4E-BP1(Thr37/46) | Cell Signalling Technology, 2855 | 1: 1500 |
| Mouse anti-H4K20me3 | Active Motif, 39671 | 1 µg/ml |
| Mouse anti-H4K20me1 | Active Motif, 39727 | 1 µg/ml |
| Rabbit anti-H3K9me3 | Active Motif, 39765 | 1 µg/ml |
| Mouse anti-H3K9me2 | Abcam, 1220 | 5 µg/ml |
| Rabbit anti-H3K27me3 | Active Motif, 39155 | 2 µg/ml |
| Mouse anti-H3K4me3 | Abcam, 12209 | 10 µg/ml |
| Rabbit anti-H3K9ac | Active Motif, 39917 | 1 µg/ml |
| Rabbit anti-H4K16ac | Abcam, 109463 | 10 µg/ml |
| Goat anti-rabbit Alexa 488 | ThermoFisher Scientific, A-11008 | 5 µg/ml |
| Goat anti-rabbit IgG-HRP | Cell Signalling Technology, 7074 | 1:200 |
| ImPRESS HRP Universal anti-mouse/rabbit IgG reagent | Vector Laboratories, MP-7500 | Ready to use |
